# When Two Lesions Collide: Coexisting Vasospasm and Atherosclerotic Disease Complicating Percutaneous Coronary Intervention in ST-Segment Elevation Myocardial Infarction

**DOI:** 10.7759/cureus.107922

**Published:** 2026-04-28

**Authors:** Eric Pin-Shiuan Chen, Boran Mao, Muhammad Rayyan Masood, Zubian Ahmed, Skylar Bentley, Sanaa Tahir, Sayed T Hussain

**Affiliations:** 1 Internal Medicine, University of Central Florida College of Medicine, Orlando, USA; 2 Medicine, Yeditepe University Faculty of Medicine, Istanbul, TUR; 3 Medicine and Surgery, Faisalabad Medical University, Faisalabad, PAK; 4 Research, Orlando College of Osteopathic Medicine, Winter Garden, USA; 5 Medicine, Orlando College of Osteopathic Medicine, Winter Garden, USA; 6 Cardiology, University of Central Florida College of Medicine, Orlando, USA

**Keywords:** acute coronary syndrome, door-to-balloon time, intravascular ultrasound (ivus), percutaneous coronary intervention, right coronary artery (rca), st-segment elevation myocardial infarction (stemi), vasospasm

## Abstract

Timely reperfusion is central to the management of ST-segment elevation myocardial infarction (STEMI), but dynamic coronary processes such as vasospasm may complicate primary percutaneous coronary intervention (PCI). We present a 50-year-old male with hyperlipidemia, active tobacco use, daily nicotine vaping, and a family history of heart disease who presented approximately three hours after the onset of persistent chest pain, following three days of intermittent left arm pain radiating to the left shoulder. Initial troponin was negative. His initial electrocardiogram was unremarkable, but repeat electrocardiography 30 minutes later demonstrated inferior ST-segment elevation, prompting emergent transfer for primary PCI. Coronary angiography demonstrated a smooth proximal right coronary artery narrowing and a separate distal stenotic lesion. The proximal narrowing resolved completely after intracoronary nitroglycerin, and intravascular ultrasound showed no plaque or thrombus at that site. ST-segment elevations did not improve after intracoronary nitroglycerin alone. Despite nitroglycerin administration, the distal lesion persisted and was treated with balloon angioplasty followed by stent implantation, with a door-to-balloon time of 117 minutes. He was discharged on vasodilator therapy (initially nitrates, later transitioned to a calcium channel blocker due to intolerance). This case highlights how coronary vasospasm may accompany fixed obstructive disease during STEMI and create additional diagnostic complexity during primary PCI, with implications for lesion assessment, avoidance of unnecessary intervention, and post-PCI vasodilator therapy.

## Introduction

Rapid identification and reperfusion are critical in ST-segment elevation myocardial infarction (STEMI), as even brief delays in door-to-balloon time adversely affect outcomes [1-3]. Although most delays arise from clinical instability or procedural challenges, uncommon dynamic factors may further complicate primary percutaneous coronary intervention (PCI). Coronary vasospasm, or variant angina, is a transient constriction of the coronary arteries that can lead to myocardial ischemia, infarction, or life-threatening arrhythmias in the absence of significant atherosclerotic disease [4-6]. It predominantly occurs in middle-aged men and may be precipitated by factors such as tobacco use, psychological stress, or exposure to certain pharmacologic agents, including cocaine, amphetamines, and triptans [7-9]. Prompt recognition is essential, as vasospasm often responds well to nitrates and calcium channel blockers and may mimic or coexist with fixed atherosclerotic disease. Angiographically, vasospasm may appear as a focal stenosis, creating diagnostic ambiguity and risking misidentification of the culprit lesion, which may lead to unnecessary stent placement. This distinction is critical, as it directly influences real-time procedural decision-making during primary PCI. Adjunctive intracoronary imaging, such as intravascular ultrasound (IVUS) or optical coherence tomography (OCT), may help differentiate dynamic vasospasm from fixed obstructive disease [10,11].

We present a case of STEMI in which a true atherosclerotic culprit lesion was accompanied by a separate proximal coronary vasospasm, resulting in dynamic angiographic findings that complicated assessment of the culprit lesion during primary PCI.

## Case presentation

A 50-year-old male presented to a free-standing emergency department approximately three hours after awakening with constant burning chest pain associated with nausea. He reported experiencing intermittent left arm pain radiating to the left shoulder for three days prior to presentation. His past medical history was notable for hyperlipidemia and a strong family history of myocardial infarction. He was an active smoker with an estimated seven pack-year smoking history and reported daily use of nicotine vaping products containing 5% nicotine. He had a remote history of cocaine use but had abstained for 18 years. He denied associated dizziness, palpitation, shortness of breath, neck or jaw pain, and had no prior similar episodes or known history of coronary artery disease. He was not taking any medications at home prior to presentation.

**Figure 1:**
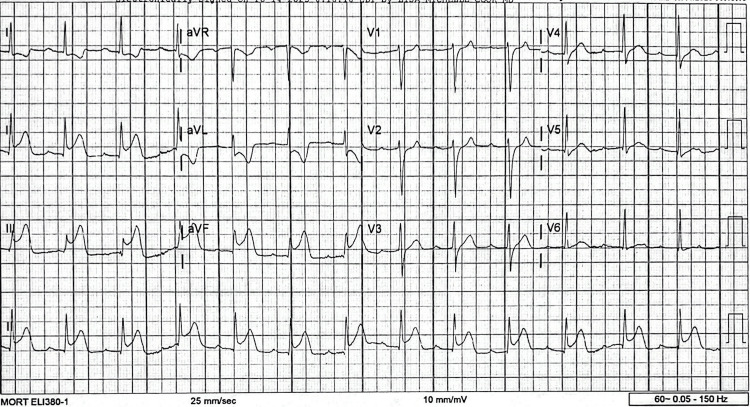
Repeat electrocardiogram demonstrating ST-segment elevation in leads II, III, and aVF, with reciprocal ST-segment depression in leads I and aVL, consistent with an acute inferior STEMI. STEMI: ST-segment elevation myocardial infarction.

On presentation, the patient was hemodynamically stable with a normal heart rate, blood pressure, and oxygen saturation. Cardiovascular examination was unremarkable, with no murmurs, gallops, or additional heart sounds appreciated. The remainder of the physical examination was unremarkable. An initial electrocardiogram (ECG) demonstrated normal sinus rhythm without ischemic changes or ST-segment abnormalities. Initial high-sensitivity troponin T was within normal limits. Hemoglobin A1c was 5.8%. Due to persistent chest pain, a repeat ECG was obtained approximately 30 minutes later, which demonstrated new ST-segment elevations in leads II, III, and aVF consistent with an acute inferior STEMI (Figure 1). A STEMI alert was activated at 07:43, and the patient was transferred emergently to a PCI-capable facility for primary PCI. He received aspirin loading and intravenous heparin prior to transfer for coronary angiography.

**Figure 2:**
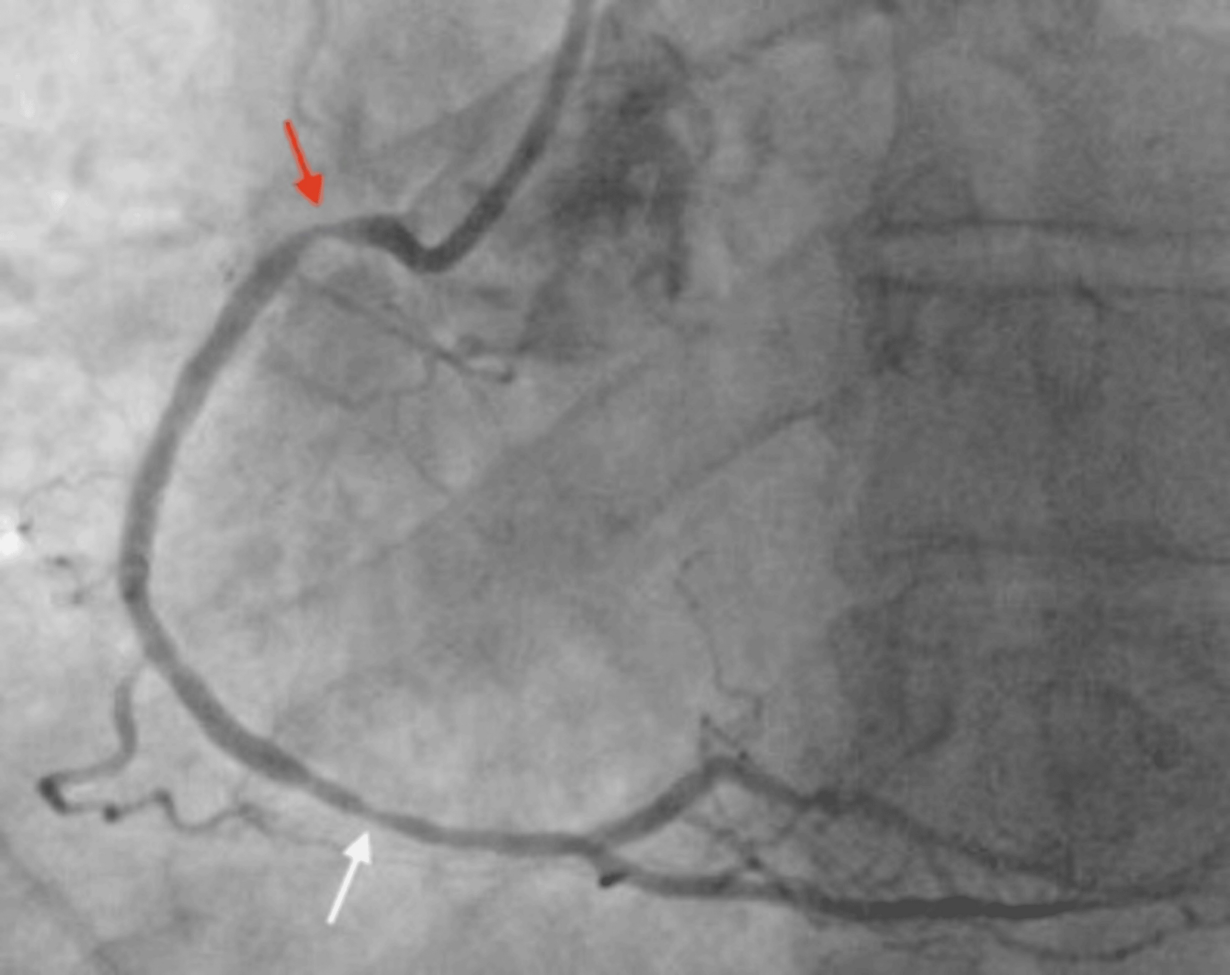
Coronary angiography demonstrating a smooth focal narrowing of the proximal right coronary artery (RCA) and a separate 70-80% distal RCA stenotic lesion. The red arrow indicates the proximal RCA narrowing, and the white arrow indicates the distal RCA lesion.

**Figure 3:**
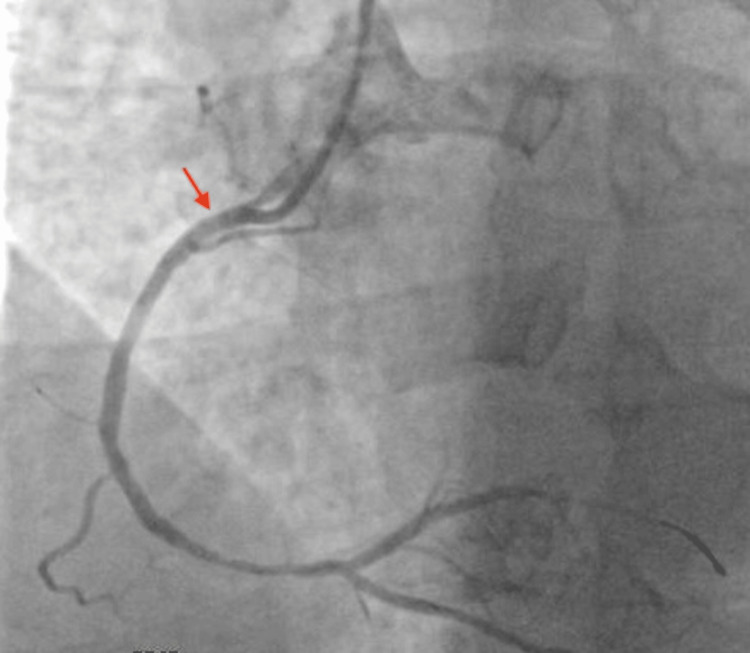
Coronary angiography demonstrating complete resolution of the proximal right coronary artery (RCA) narrowing after intracoronary nitroglycerin administration, supporting a vasospastic rather than fixed obstructive lesion. The red arrow indicates the previously narrowed proximal RCA segment.

**Figure 4:**
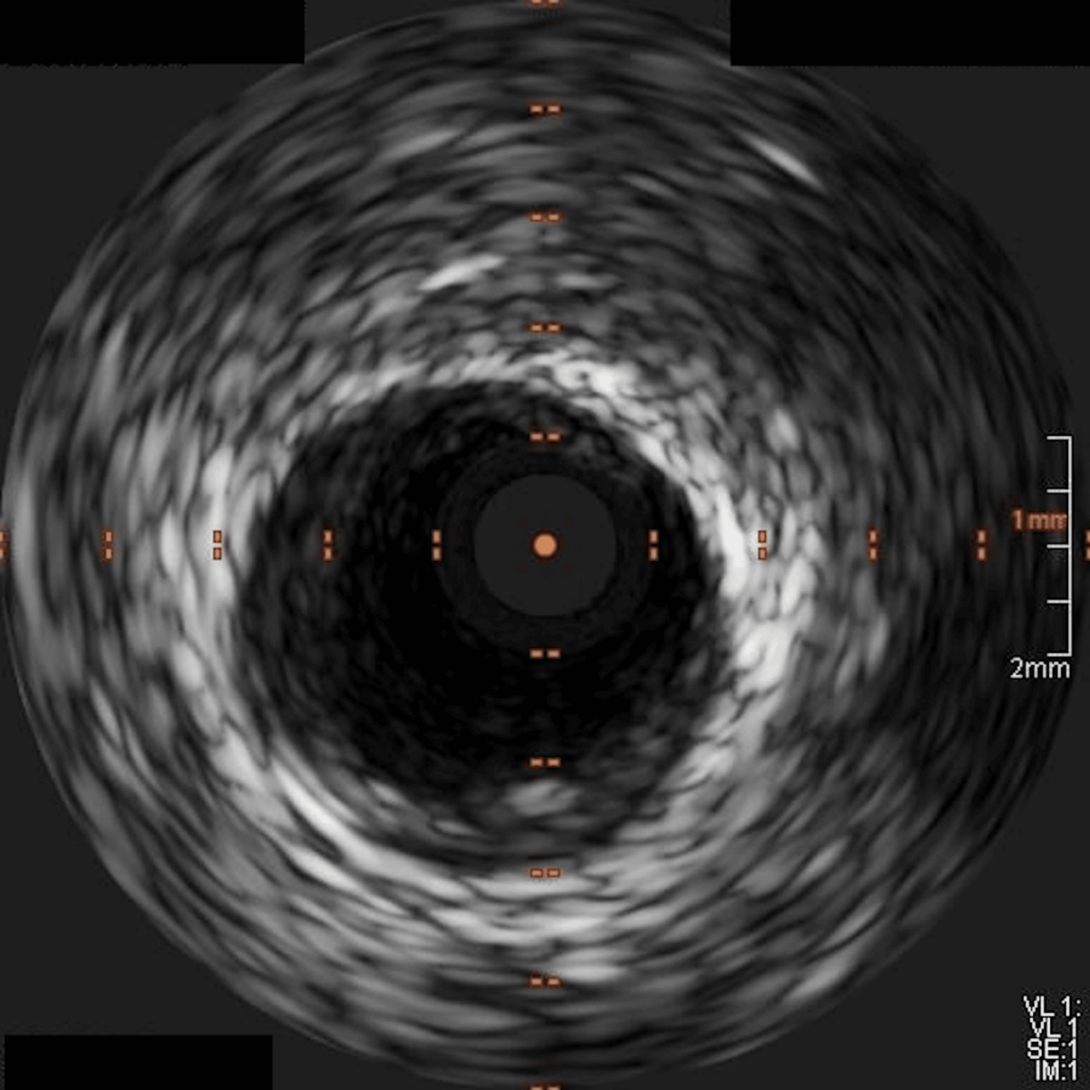
Intravascular ultrasound (IVUS) of the proximal right coronary artery after intracoronary nitroglycerin administration demonstrating minimal plaque burden and no thrombus, supporting coronary vasospasm rather than a fixed atherosclerotic lesion.

Coronary angiography was initiated at 09:10. Coronary angiography of the right coronary artery (RCA) demonstrated a smooth 90% focal narrowing in the proximal segment and 70-80% stenosis in the distal segment (Figure 2). The left coronary system revealed no significant disease in the left anterior descending artery and 30-40% stenosis in the left circumflex artery. The differential diagnosis for the proximal RCA narrowing included vasospasm, fixed stenosis, and thrombus. Given the smooth appearance of the proximal narrowing, intracoronary nitroglycerin was administered at 09:28, resulting in complete angiographic resolution of the proximal RCA narrowing approximately two minutes later (Figure 3). ST-segment elevations did not improve after intracoronary nitroglycerin alone. In contrast, the distal RCA stenosis persisted despite nitroglycerin administration, supporting a fixed atherosclerotic lesion as the likely culprit for the myocardial infarction. Intravascular ultrasound (IVUS) of the proximal RCA performed between 09:31 and 09:33 demonstrated minimal plaque burden without thrombus, confirming a vasospastic mechanism (Figure 4). In contrast, IVUS findings at the distal RCA were consistent with approximately 80% stenosis, diffuse plaque burden, and mild punctate nonconcentric calcification, supporting underlying atherosclerotic disease. Balloon angioplasty of the distal RCA lesion was performed at 09:39, achieving successful revascularization, followed by stent implantation in the distal RCA with restoration of Thrombolysis In Myocardial Infarction (TIMI) 3 flow. The patient’s chest pain improved following revascularization, with mild residual left arm discomfort. A repeat ECG obtained at 10:42 demonstrated resolution of ST-segment elevations. Transthoracic echocardiography performed after coronary angiography demonstrated preserved left ventricular systolic function, with an ejection fraction of 60-65% and no regional wall motion abnormalities.

The patient’s hospital course was uncomplicated. He was discharged on dual antiplatelet therapy with aspirin 81 mg daily and prasugrel 10 mg daily, as well as carvedilol 3.125 mg twice daily, isosorbide mononitrate extended-release 30 mg daily, and rosuvastatin 40 mg daily. At one-week follow-up, the patient reported persistent headaches attributed to nitrate therapy. As a result, isosorbide mononitrate was discontinued and replaced with nifedipine extended-release 30 mg daily for ongoing management of coronary vasospasm. The patient remained clinically stable without recurrent symptoms on follow-up. Longer-term follow-up was not available.

## Discussion

Current American College of Cardiology (ACC)/American Heart Association (AHA) guidelines recommend achieving a door-to-balloon time of ≤90 minutes for STEMI patients treated at PCI-capable hospitals and ≤120 minutes from first medical contact for those requiring transfer [1]. Each 30-minute delay in reperfusion increases the relative risk of one-year mortality by approximately 7.5% [12]. The most common causes of delayed door-to-balloon time include cardiac arrest requiring intubation, atypical presentation, emergency department workflow delays, technical difficulty crossing the culprit lesion, challenging vascular access, delayed consent, and off-hours presentation [3]. However, delays may also arise from less common mechanisms, including dynamic coronary processes such as vasospasm.

Coronary artery spasm is characterized by a marked (>90%) constriction of an epicardial coronary artery, leading to a significant reduction in myocardial blood flow [4]. In patients with myocardial infarction with nonobstructive coronary arteries (MINOCA), coronary vasospasm has been identified in nearly half of cases undergoing provocative testing, as demonstrated in a prior study [13]. The typical presentation of coronary vasospasm consists of recurrent rest angina, often in the early morning, that resolves rapidly with short-acting nitrates. Episodes are usually accompanied by transient ischemic ECG changes, most commonly ST-segment elevation, but may also manifest as ST depression or new negative U waves in contiguous leads [14]. Angiographically, the hallmark of vasospasm is a severe, transient narrowing that resolves promptly with nitrate administration, whether observed spontaneously or during provocative testing, and most often occurs at branch points or non-plaque sites [4,14,15]. Prompt recognition is crucial, as early treatment effectively prevents recurrence and reduces major adverse cardiac events [5,16,17]. Short-acting nitrates provide prompt resolution of acute episodes, though the long-term benefits of sustained nitrate therapy remain unclear [4,18]. Calcium channel blockers are the primary agents for long-term management, effectively reducing anginal frequency and preventing recurrent ischemic events [4,17,19]. Both dihydropyridine and nondihydropyridine calcium channel blockers are equally effective as first-line therapies [20].

In the present case, the proximal RCA vasospasm was most likely an epiphenomenon rather than the primary infarct mechanism. Although the proximal narrowing resolved promptly with intracoronary nitroglycerin, the ST-segment elevations did not improve after nitroglycerin alone, the distal RCA lesion persisted despite vasodilator administration, and ECG resolution occurred only after distal lesion revascularization. Together, these findings support the distal RCA lesion as the culprit lesion and the proximal vasospasm as a concurrent dynamic process that complicated angiographic interpretation rather than the primary cause of infarction. The distal IVUS findings, including approximately 80% stenosis, diffuse plaque burden, and mild punctate nonconcentric calcification, further supported structurally significant atherosclerotic disease. However, the available imaging did not allow definitive classification of plaque rupture versus plaque erosion.

This distinction was clinically important because misinterpreting vasospasm as fixed obstructive disease could have led to unnecessary stenting of the proximal RCA. Such an error would have increased procedural complexity and permanent stent burden without addressing the true culprit lesion. In this case, intracoronary nitroglycerin and IVUS were added an estimated 10-15 minutes before definitive treatment of the distal lesion. For a transferred STEMI patient, this represents approximately 8%-13% of the 120-minute first-medical-contact-to-device benchmark and consumed much of the remaining time margin for reperfusion. Thus, even a relatively brief delay may be clinically relevant when transport time has already used a substantial portion of the recommended reperfusion window.

Although coronary vasospasm may coexist with atherosclerotic disease, this case is notable because a nitrate-responsive proximal vasospasm was present alongside a separate fixed distal culprit lesion during primary PCI [21]. This created real-time diagnostic ambiguity in the cath lab and required immediate distinction between dynamic obstruction and true obstructive coronary disease. The practical teaching point is not only that vasospasm can mimic fixed stenosis angiographically, but also that it can alter lesion prioritization, prolong decision-making, and expose patients to unnecessary intervention if interpreted incorrectly.

Catheter-induced spasm was considered but was felt to be less likely. Catheter-associated spasm most commonly involves the RCA but remains uncommon overall, occurring in approximately 0.75% of cases [22]. In general, catheter-induced spasm occurs at the catheter tip and is temporally related to catheter engagement or manipulation [23]. In this case, the narrowing was not confined to the catheter tip, did not demonstrate a clear temporal relationship to catheter manipulation, and resolved after intracoronary nitroglycerin rather than catheter repositioning alone, making a spontaneous focal vasospastic process more likely.

IVUS was particularly useful because it could be performed rapidly in the acute PCI setting and helped distinguish a dynamic vasospastic segment from fixed atherosclerotic disease [10,24]. At the proximal RCA, IVUS demonstrated minimal plaque burden and no thrombus, supporting vasospasm rather than structural obstructive disease. At the distal RCA, IVUS showed atherosclerotic plaque and persistent severe stenosis, supporting culprit-lesion treatment. OCT could potentially have provided more detailed superficial plaque characterization, including better assessment of plaque rupture or erosion [24], but it was not necessary for immediate lesion discrimination once the proximal segment normalized with nitroglycerin and IVUS excluded significant plaque or thrombus proximally. In addition, OCT would have required additional contrast and blood clearance during an acute STEMI intervention.

The discharge regimen also warrants brief comment. Although beta-blockers may theoretically exacerbate vasospastic angina in some settings, carvedilol was prescribed here in the context of post-myocardial infarction secondary prevention for a patient with a fixed culprit lesion [25]. Vasospasm-directed therapy was addressed separately with nitrates initially and then transitioned to a calcium channel blocker after nitrate intolerance.

In summary, vasospasm can coexist with fixed atherosclerotic disease and may create dynamic obstruction that delays reperfusion during primary PCI. Clinicians should consider vasospasm in patients who fit the typical demographic profile, such as middle-aged males and individuals who smoke or have other vasoconstrictive triggers, particularly when angiographic narrowing appears disproportionate, changes after nitrate administration, or occurs at non-culprit segments. Timely recognition of this dynamic process can help prevent further delay in revascularization.

## Conclusions

Coronary vasospasm can occur during primary PCI and may create significant diagnostic and procedural complexity when it coexists with fixed obstructive coronary disease. Clinicians should consider vasospasm in patients who fit the typical demographic profile, such as middle-aged men and individuals who smoke or have other vasoconstrictive triggers, particularly when angiographic narrowing appears smooth, occurs in a separate coronary segment, or improves after intracoronary nitroglycerin. In ambiguous lesions, early administration of intracoronary nitrates and, when uncertainty persists, adjunctive intravascular imaging such as IVUS may help distinguish dynamic vasospasm from fixed atherosclerotic disease. This distinction is important to avoid unnecessary PCI in lesions that may not represent the true culprit site while ensuring timely treatment of the actual culprit lesion. Long-term management should also address vasospasm-directed therapy, particularly calcium channel blockers, along with smoking cessation and other risk factor modifications.

## References

[1] Rao SV, O'Donoghue ML, Ruel M (2025). 2025 ACC/AHA/ACEP/NAEMSP/SCAI guideline for the management of patients with acute coronary syndromes: a report of the American College of Cardiology/American Heart Association Joint Committee on clinical practice guidelines. Circulation.

[2] Park J, Choi KH, Lee JM (2019). Prognostic implications of door-to-balloon time and onset-to-door time on mortality in patients with ST-segment-elevation myocardial infarction treated with primary percutaneous coronary intervention. J Am Heart Assoc.

[3] Jollis JG, Granger CB, Zègre-Hemsey JK (2022). Treatment time and in-hospital mortality among patients with ST-segment elevation myocardial infarction, 2018-2021. JAMA.

[4] Tamis-Holland JE, Jneid H, Reynolds HR (2019). Contemporary diagnosis and management of patients with myocardial infarction in the absence of obstructive coronary artery disease: a scientific statement from the American Heart Association. Circulation.

[5] Sandau KE, Funk M, Auerbach A (2017). Update to practice standards for electrocardiographic monitoring in hospital settings: a scientific statement from the American Heart Association. Circulation.

[6] Kusama Y, Kodani E, Nakagomi A, Otsuka T, Atarashi H, Kishida H, Mizuno K (2011). Variant angina and coronary artery spasm: the clinical spectrum, pathophysiology, and management. J Nippon Med Sch.

[7] Montone RA, Niccoli G, Russo M (2020). Clinical, angiographic and echocardiographic correlates of epicardial and microvascular spasm in patients with myocardial ischaemia and non-obstructive coronary arteries. Clin Res Cardiol.

[8] Morita S, Mizuno Y, Harada E (2014). Differences and interactions between risk factors for coronary spasm and atherosclerosis--smoking, aging, inflammation, and blood pressure. Intern Med.

[9] Rallidis LS, Xenogiannis I, Brilakis ES, Bhatt DL (2022). Causes, angiographic characteristics, and management of premature myocardial infarction: JACC State-of-the-Art Review. J Am Coll Cardiol.

[10] Hong YJ, Jeong MH, Choi YH (2010). Plaque components at coronary sites with focal spasm in patients with variant angina: virtual histology-intravascular ultrasound analysis. Int J Cardiol.

[11] Tanaka A, Shimada K, Tearney GJ (2011). Conformational change in coronary artery structure assessed by optical coherence tomography in patients with vasospastic angina. J Am Coll Cardiol.

[12] De Luca G, Suryapranata H, Ottervanger JP, Antman EM (2004). Time delay to treatment and mortality in primary angioplasty for acute myocardial infarction: every minute of delay counts. Circulation.

[13] Montone RA, Niccoli G, Fracassi F (2018). Patients with acute myocardial infarction and non-obstructive coronary arteries: safety and prognostic relevance of invasive coronary provocative tests. Eur Heart J.

[14] Virani SS, Newby LK, Arnold SV (2023). 2023 AHA/ACC/ACCP/ASPC/NLA/PCNA guideline for the management of patients with chronic coronary disease: a report of the American Heart Association/American College of Cardiology joint committee on clinical practice guidelines. Circulation.

[15] Nakagawa H, Morikawa Y, Mizuno Y (2009). Coronary spasm preferentially occurs at branch points: an angiographic comparison with atherosclerotic plaque. Circ Cardiovasc Interv.

[16] Al-Khatib SM, Stevenson WG, Ackerman MJ (2018). 2017 AHA/ACC/HRS guideline for management of patients with ventricular arrhythmias and the prevention of sudden cardiac death: a report of the American College of Cardiology/American Heart Association Task Force on Clinical Practice Guidelines and the Heart Rhythm Society. Heart Rhythm.

[17] Beltrame JF (2022). Management of vasospastic angina. Heart.

[18] Beltrame JF, Crea F, Kaski JC (2016). The who, what, why, when, how and where of vasospastic angina. Circ J.

[19] Van Spall HG, Overgaard CB, Abramson BL (2005). Coronary vasospasm: a case report and review of the literature. Can J Cardiol.

[20] Higuma T, Oikawa K, Kato T (2010). Comparison of the effects of long-acting nifedipine CR and diltiazem R in patients with vasospastic angina: Aomori coronary spastic angina study. J Cardiol.

[21] Peeters D, Woelders E, Jansen T (2025). Association between coronary artery spasm and atherosclerotic disease. JACC Cardiovasc Imaging.

[22] Sueda S, Fujimoto K, Sasaki Y, Sakaue T, Habara H, Kohno H (2019). Catheter-induced spasm in the proximal right coronary artery. Intern Med.

[23] Friedman AC, Spindola-Franco H, Nivatpumin T (1979). Coronary spasm: Prinzmetal's variant angina vs. catheter-induced spasm; refractory spasm vs. fixed stenosis. AJR Am J Roentgenol.

[24] Truesdell AG, Alasnag MA, Kaul P (2023). Intravascular imaging during percutaneous coronary intervention: JACC State-of-the-Art Review. J Am Coll Cardiol.

[25] Kern MJ, Ganz P, Horowitz JD (1983). Potentiation of coronary vasoconstriction by beta-adrenergic blockade in patients with coronary artery disease. Circulation.

